# Twelfth thoracic vertebra erector spinae plane block for postoperative analgesia and early recovery after lumbar spine surgery in elderly patients: a single-blind randomized controlled trial

**DOI:** 10.1186/s12871-023-02351-2

**Published:** 2023-12-07

**Authors:** Aijia Zhang, Jiaxin Chen, Xiaoyun Zhang, Tao Jiang, Dongmei Li, Xuemin Cai, Haixu Wang, Wengang Ding

**Affiliations:** 1 Department of Anesthesiology, The Second Affiliated Hospital of Kunming Medical University, Kunming, Yunnan China; 2 Department of Anesthesiology, Women’s Hospital of Zhejiang University, Hangzhou, Zhejiang China; 3 Department of Anesthesiology, The Second Affiliated Hospital of Harbin Medical University, 246 Xuefu Road, Harbin, Heilongjiang, 150086 Heilongjiang China; 4 Department of Anesthesiology, Nanchong Central Hospital, Nanchong, Sichuan China

**Keywords:** Lumbar spine Surgery, Ultrasound-guided erector spinae plane block, Postoperative pain, Postoperative delirium, Elderly patients

## Abstract

**Background:**

Severe pain after lumbar spine surgery can delay recovery in elderly patients. We explored the efficacy of T12 erector spinal plane block (ESPB) in elderly patients who underwent lumbar spine surgery.

**Methods:**

A total of 230 patients undergoing lumbar spine surgery were divided and randomly allocated to ultrasound-guided ESPB (n = 115) and control (n = 115) groups. The ESPB group received 20 mL of 0.4% ropivacaine bilaterally at the T12 level after intubation, whereas the control group did not receive a block. The primary outcome was the numeric rating scale (NRS) score at 12 h after surgery. Secondary outcomes included the NRS score and tramadol use within 72 h postoperatively, intraoperative remifentanil use, incidence of postoperative delirium (POD), complications of ESPB, ambulation time, and length of hospitalization after surgery.

**Results:**

The12-hour NRS (median (IQR)) score was remarkably lower in the ESPB group than in the control group (2 (1–3) vs. 3 (2–4), p = 0.004), as well as NRS score within 48 h (P < 0.01). The ESPB group had less intraoperative remifentanil use (P < 0.001), and less tramadol use within 72 h postoperatively (P < 0.001). Seven patients (6.7%) developed POD in the ESPB group and ten patients (9.3%) in the control group, without any statistically significant difference (P > 0.05). The ambulation time and length of hospitalization after surgery were shorter in the ESPB group than in the control group (P < 0.05). No ESPB-related complications were observed.

**Conclusions:**

Bilateral T12 ESPB lowered the NRS score within 48 h after lumbar spine surgery, decreased perioperative opioid use and resulted in faster recovery in elderly patients but did not significantly reduce the incidence of POD.

**Trial registration:**

The study was retrospectively registered at www.chictr.org.cn (ChiCTR2100042037) on January 12, 2021.

## Introduction

As the population ages, more older people are undergoing lumbar spine surgery. Posterior lumbar spine surgery is usually an open procedure that results in considerable postoperative pain due to paraspinal muscle dissection and disc removal. In a comprehensive analysis of 179 postoperative pain types, spinal surgery pain ranked second after open calcaneal surgery [1]. Postoperative pain, as a result of lumbar spine surgery, commonly lasts more than three days, with the most severe pain occurring in the first four hours and progressively diminishing after the third day. Severe postoperative pain can cause depressive episodes, sleep disturbances, and ambulation delays, all of which decrease postoperative comfort and delay recovery. Postoperative pain may also increase the risk of cardiac ischemia and the incidence of postoperative delirium (POD) [2] in elderly patients.

Opioids are the main analgesic drugs used after spinal surgery. Due to reduced organ function, elderly patients are less tolerant of opioids and are more susceptible to respiratory depression, excessive sedation, and other adverse effects. Additionally, neuraxial blocks are rarely utilized because they block the anterior branches of the spinal nerves and interfere with intraoperative monitoring of lower limb motor function. With the development of ultrasound visualization techniques, regional block techniques play an increasingly important role in multimode analgesia. An erector spinae plane block (ESPB) is a truncal block in which a local anesthetic is injected between the transverse process and the erector spinae muscle (ESM) under ultrasound guidance. Local anesthetics are widely diffused between the transverse process and the ESM, thus blocking the dorsal branches of spinal nerves at multiple levels [3]. Numerous researchers have reported the good analgesic effect of lumbar ESPB in spinal surgery. However, few researchers have reported the application of low-thoracic ESPB during lumbar spine surgery [4–6].

A single-blind randomized controlled trial was designed to principally evaluate the impact of T12 ESPB on postoperative pain scores in elderly patients who underwent lumbar spine surgery. Second, the effects of T12 ESPB on intraoperative hemodynamics, perioperative opioid use, and recovery outcomes were also investigated.

## Methods

This prospective, randomized, single-blinded trial protocol was approved by the Ethics Committee of the Second Affiliated Hospital of Harbin Medical University on November 10, 2020 (KY2020-222), and it was registered at www.chictr.org.cnon12/01/2021 (registration No.: ChiCTR2100042037). The methodology of this study follows the Helsinki Declaration (revised, 2014). From November 10, 2020, to May 30, 2021, 230 patients were selected for posterior lumbar decompression or fusion surgery. All patients signed written informed consent before enrollment. Inclusion criteria: age ≥ 60 years, BMI ≤ 32 kg/m^2^, and American Society of Anesthesiologists (ASA) physical status I to III. The exclusion criteria were as follows: (1) schizophrenia, epilepsy or Parkinson’s disease; (2) inability to communicate or have cognitive dysfunction (Screening of mini-Mental State Examination (MMSE)) [7]; (3) history of traumatic brain injury, stroke or neurosurgery; (4) injection site infection; or (5) contraindications to flurbiprofen. Exit criteria included (1) withdrawal or early discharge from the study; (2) self-administered analgesics; (3) cerebrospinal fluid leaking during surgery; or (4) serious cardiac and cerebrovascular events during the trial.

### Randomization and blinding

The anesthesiologist in charge of intraoperative management evaluated the patients on the day before the operation and allocated them to the ESPB or control group based on a 1:1 random number (SPSS Statistics 26.0, IBM, USA). Standard general anesthesia was conducted in the two groups. The ESPB group received T12 ESPB after intubation, whereas the control group did not receive the intervention. T12 ESPB was performed by a specialist who was experienced in nerve blocks. Patients, postoperative physicians and postoperative investigators were unaware of the group assignments throughout the trial.

### Perioperative management

The electrocardiogram (ECG), pulse oxygen saturation, and bispectral index (BIS) were monitored once the patient entered the operating room. Depending on the patient’s condition, invasive or noninvasive arterial blood pressure monitoring was performed. Intravenous (IV) sufentanil (0.3 µg/kg), etomidate (0.2–0.3 mg/kg), and atracurium (0.6 mg/kg) were used to induce general anesthesia. Following intubation, sevoflurane (1.5-3%) was inhaled to maintain BIS values between 40 and 60. Intermittent atracurium injections kept muscles relaxed, and the continuous infusion volume of remifentanil (0.1–0.2 µg/(kg·min)) was adjusted to maintain the heart rate (HR) and the mean arterial blood pressure (MAP) within 80–120% of baseline. When the MAP was less than 80% of the baseline value, IV ephedrine or IV phenylephrine was administered, and IV atropine was administered when the HR was less than 40 bpm. IV 5 mg dexamethasone were administered for PONV prevention. Flurbiprofen (100 mg) was injected intravenously 30 min before the end of the operation to ensure postoperative analgesia was achieved. When the patient was awake and the tidal volume was sufficient, the tracheal tube was removed. The anesthesiologist then transferred the patient to the postanesthesia care unit (PACU).

### Ultrasound-guided bilateral block

**Fig. 1:**
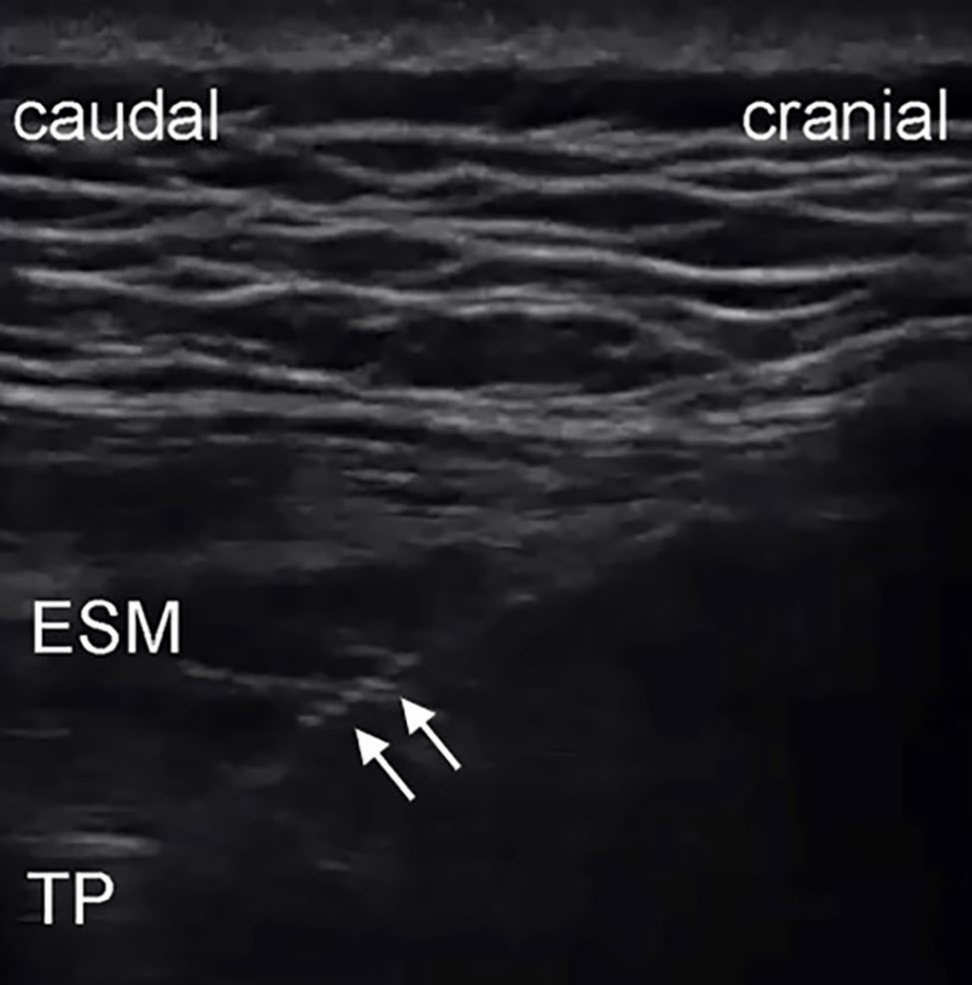
Ultrasound images of ESPB with the in-plane technique. Arrows, needle shaft. Abbreviations: ESM, erector spinae muscle; TP, transverse process

After induction, the patient was changed to a prone position. The skin was disinfected with iodophor, and the high-frequency linear ultrasound probe (6–13 Mhz, SonoSite S Series, USA) was placed in a sterile sleeve. The probe was placed longitudinally at the 12th rib of the midscapular line and moved inward along the 12th rib until the tip of the transverse process was reached. Using an in-plane method, the needle (0.71 × 120 mm, 22 G, B Braun, Germany) was inserted into the skin at the head of the probe until it reached the transverse process. Two milliliters of normal saline (NaCl 0.9%) were injected to identify the needle tip position. If the needle tip was located between the transverse process and the ESM and was not in the muscle, 20 mL 0.4% ropivacaine (AstraZeneca AB, Sweden) was injected immediately (Fig. 1). The procedure was then repeated on the opposite side.

### Postoperative pain management

Both groups were administered intravenously guttae flurbiprofen 100 mg twice a day to maintain postoperative analgesia. When the numeric rating scale (NRS) score was ≥ 4, an intramuscular injection of 100 mg tramadol was administered.

### Data collection

​ Intraoperative data were collected, including the duration of surgery, remifentanil use, total blood loss, hemodynamic parameters, and vasoactive medication dosage. Postoperative data included extubation time, Sedation-Agitation Scale (SAS) score [8] after extubation, NRS score (evaluated at 0, 0.5, 4, 12, 24, 36, 48, 60, and 72 h postoperatively), tramadol use within 72 h postoperatively, incidence of POD within 3 days after surgery (POD diagnosed by Confusion Assessment Method (CAM)) [9], postoperative nausea and vomiting (PONV), ESPB-related complications, ambulation time (the period between the completion of surgery until and when the patient could stand up and walk with support from a family member), and length of hospitalization after surgery.

Intraoperative data were collected by the anesthesiologists and postoperative data were obtained by the trained investigators.

### Outcomes

The primary outcome was the NRS pain score (0–10; 0, no pain; 1–3, mild pain; 4–6, moderate pain; 7–10, severe pain) at 12 h after surgery. Secondary outcomes included the NRS pain score and tramadol use within 72 h postoperatively, intraoperative remifentanil use, hemodynamic parameters, extubation time, SAS score after extubation, incidence of POD, incidence of PONV, ESPB-related complications, ambulation time, and length of hospitalization after surgery.

### Statistical analysis

The sample size was estimated with the MedSci Application (V.6.2.2, MedSci, China). We performed sample size calculations, in which a 1-point reduction in the NRS score at 12 h was considered clinically relevant. Eight patients in each group were included in our pilot study. The NRS score was 2 (1) (mean (SD)) in the ESPB group and 2.5 (1.4) in the control group at 12 h. According to a two-sided unpaired t test, 90 patients were required in each group to achieve 80% response and an α threshold of 0.05. Due to the nonparametric estimation parameter data, the sample size was increased by 15%, resulting in 104 patients per group. Considering loss to follow-up, we selected 115 patients per group. Statistical tests were conducted with IBM SPSS Statistics 26.0 (IBM, USA). The Shapiro―Wilk test was employed to test for normal distribution; the Student’s t test and the Mann―Whitney U test were used for continuous variables (normal and nonnormal distribution);and the Fisher’s exact or χ2 tests were used for categorical variables and proportions. A two-tailed, *P value < 0.05* was deemed statistically significant.

## Results

**Fig. 2:**
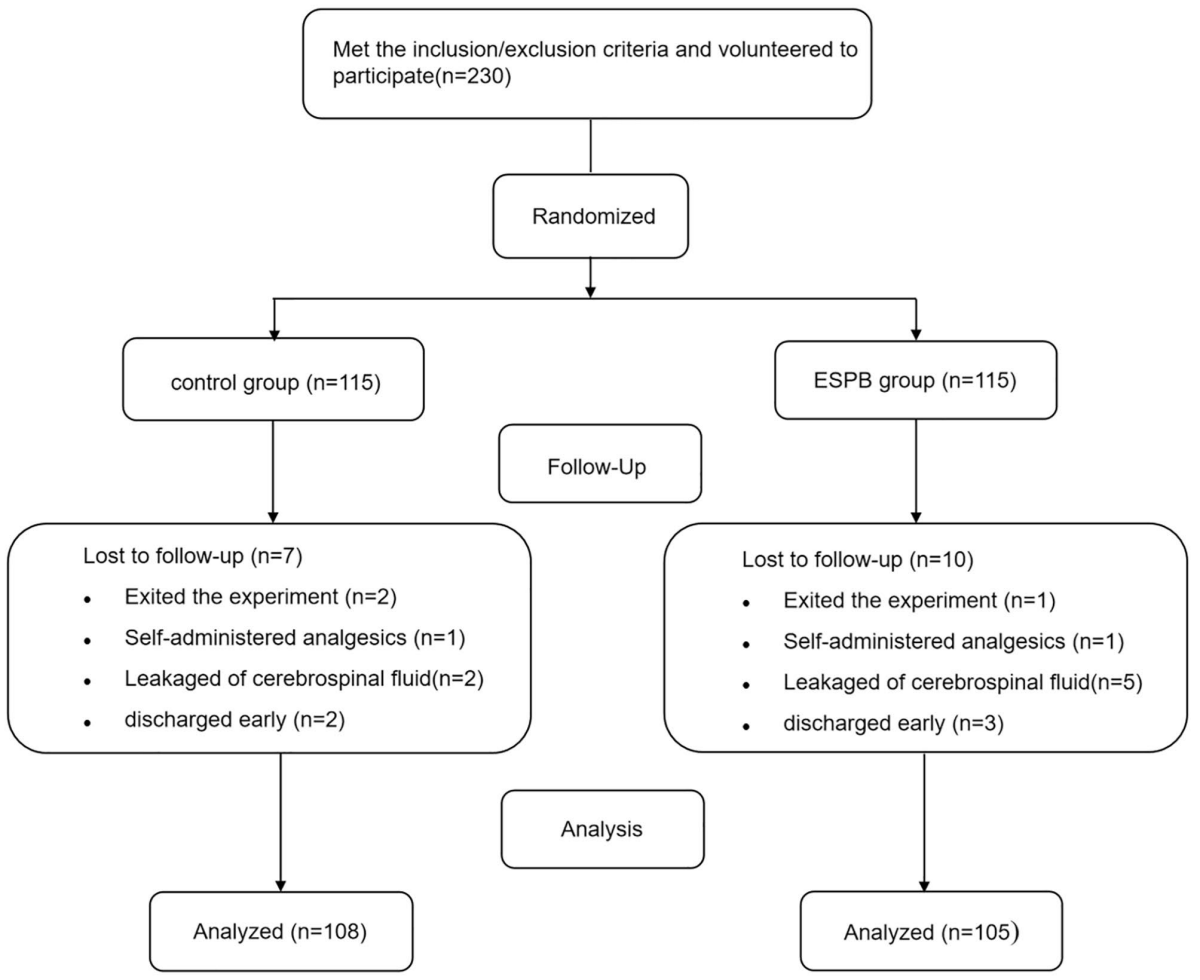
Flow chart of included patient numbers

From November 10, 2020, to May 30, 2021, 230 patients met the requirements after being screened. Three patients requested removal from the study after surgery; two patients self-administered analgesics; seven patients experienced cerebrospinal fluid leakage during the operation and the postoperative ambulation time was prolonged; and five patients were discharged early. Finally, the data of 213 patients were analyzed. Figure 2 shows the research flow chart.

### Pre- and intraoperative data

The demographic and clinical variables were comparable between the two groups (Table 1). The operative segments, operative duration, total blood loss, and use of ephedrine and phenylephrine were similar between the two groups, and the amount of remifentanil in the ESPB group was remarkably lower than that in the control group (6.67 (1.18) vs. 10.01 (1.23) µg/(kg·h), P < 0.001) (Table 1). The MAP was lower in response to the skin incision (83 (9) vs. 86 (10) mmHg, P = 0.03) in the ESPB group (Table 2).

**Table 1:** Demographic and intraoperative data

	Control group	ESPB group	p value
Sample size, n	108	105	
Age (years; mean (SD))	66.7 (5.5)	65.7 (4.4)	0.17
Sex (n (% men)	53 (49.1)	44 (41.9)	0.29
BMI (kg/m^2^; mean (SD))	24.8 (3.2)	24.7 (2.6)	0.71
Hypertension (n (%))	40(37.0)	35(33.3)	0.57
Diabetes (n (%))	37(34.3)	32(30.5)	0.56
ASA status (n (%))			0.49
I	27(25.0)	34(32.4)	
II	56(51.9)	50(47.6)	
III	25(23.1)	21(20.0)	
MMSE (mean (SD))	26.7 (2.0)	26.6 (2.0)	0.78
Operative segments (n (%))			0.87
1	34(31.5)	38(36.2)	
2	52(48.1)	49(46.6)	
3	19(17.6)	15(14.3)	
4	3(2.8)	3(2.9)	
Operative duration (min; mean (SD))	152.0 (54.5)	142.7 (45.6)	0.18
Total blood loss (mL; median (IQR))	225(100–400)	200 (100–300)	0.13
Ephedrine (mg; median (IQR))	18(10–24)	18(6–24)	0.74
Phenylephrine (µg; median (IQR))	100(0-857)	60(0-457)	0.47
Remifentanil [µg/(kg·h); mean (SD)]	10.01 (1.23)	6.67 (1.18)	< 0.001

Student’s t test or Mann―Whitney U test for continuous variables; Fisher’s exact or χ2 tests for categorical variables and proportions

ASA, American Society of Anesthesiologist; BMI, body mass index; MMSE, Mini-mental state examination; n, number; SD, standard deviation

**Table 2:** Hemodynamic parameters

	Control group	ESPB group	p value
Sample size, n	108	105	
MAP (mmHg; mean (SD))			
Upon entry into the operating room (T1)	109 (13)	111 (11)	0.17
Before skin incision (T2)	83 (8)	82(9)	0.19
At skin incision (T3)	86 (10)	83 (9)	0.03
1 h after skin incision (T4)	82 (8)	82 (7)	0.62
Before extubation (T5)	95 (10)	98 (11)	0.11
5 min after extubation (T6)	105 (9)	104(10)	0.71
h (bpm; mean (SD))			
Upon entry into the operating room (T1)	76 (11)	77 (11)	0.68
Before skin incision (T2)	60 (7)	60 (9)	0.90
At skin incision (T3)	61 (7)	61 (8)	0.85
1 h after skin incision (T4)	60 (6)	61 (7)	0.71
Before extubation (T5)	71 (13)	70 (10)	0.40
5 min after extubation (T6)	86 (10)	84 (10)	0.35

Student’s t test for continuous variables; HR: heart rate; MAP, mean arterial pressure; n, number; SD, standard deviation

### Postoperative data

**Fig. 3:**
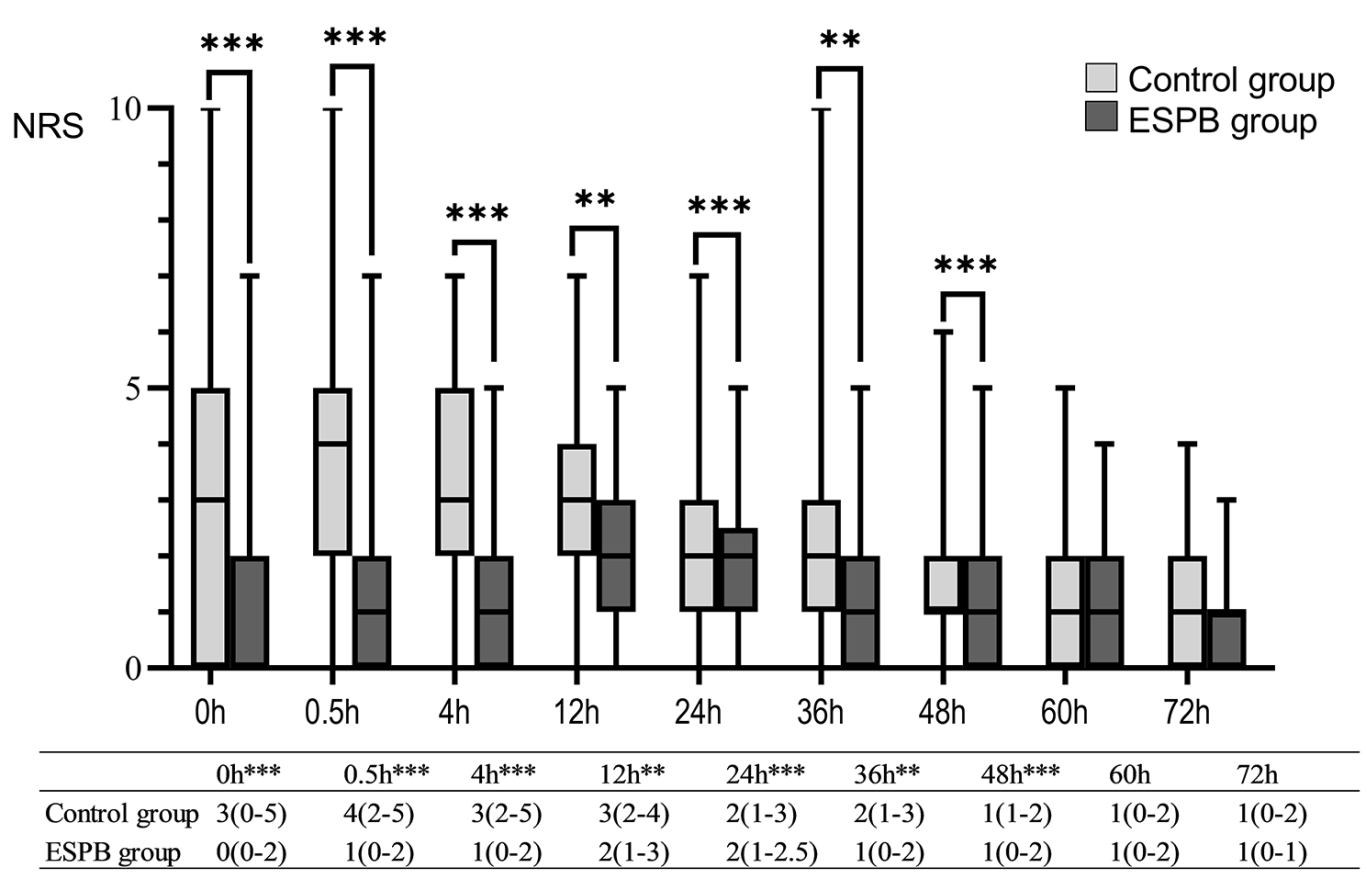
NRS pain scores (median (IQR)) within 72 h after surgery. MannWhitney U Test. ** p < 0.01, *** p < 0.001. NRS, numeric rating scales

Patients in the ESPB group had lower NRS scores at 12 h postoperatively than the control patients (2 (1, 3) vs. 3 (2–4), p < 0.01). Similar differences were observed in the pain scores at 0, 0.5, 4, 24, 36, and 48 h. In contrast, the two groups had similar NRS pain scores at 60 and 72 h postoperatively (Fig. 3). The cumulative dosage of tramadol in the ESPB group was significantly lower within 72 h after surgery (0 (0-100) vs. 100 (0-300) mg, p < 0.001). The incidence of POD was similar between groups (7 (6.7%) vs. 10 (9.3%), p = 0.49). Ambulation time in the ESPB group was 15.2 h earlier than in the control group (68.6 (24.5) vs. 83.8 (44.0) h, p < 0.01), and the length of hospitalization after surgery was 1 day shorter (9.3 (2.8) vs. 10.3 (3.2) d, p = 0.02). Significant differences were observed for extubation time, SAS score after extubation, and incidence of PONV (Table 3). No complications related to ESPB block were observed.

**Table 3:** Postoperative recovery outcomes

	Control group	ESPB group	p value
Sample size, n	108	105	
Extubation time (s; mean (SD))	772.6 (174.9)	698.5 (160.5)	< 0.001
SAS score after extubation (mean (SD))	4.3 (0.8)	3.8 (0.5)	< 0.001
PONV (n (%))	38(35.2%)	23(21.9%)	0.03
POD (n (%))	10(9.3%)	7(6.7%)	0.49
Tramadol (mg; (median (IQR))	100 (0-300)	0 (0-100)	< 0.001
Ambulation time (h; mean (SD))	83.8 (44.0)	68.6 (24.5)	< 0.01
Length of hospitalization after surgery (d; mean (SD))	10.3 (3.2)	9.3 (2.8)	0.02

Student’s t test or Mann―Whitney U test for continuous variables; Fisher’s exact or χ2 tests for categorical variables and proportions

n, number; PONV: postoperative nausea or vomiting; POD: postoperative delirium; SAS, Sedation-Agitation Scale; SD, standard deviation

## Discussion

The findings of this randomized, single-blind study showed that T12 ESPB could reduce NRS scores within 48 h postoperatively and perioperative opioid use. Compared with no block, T12 ESPB significantly shortened ambulation time and hospital stay.

ESPB was proposed for the treatment of thoracic neuropathic pain in 2016 [10]. Its extensive block of the spinal nerve’s dorsal rami is preferred by nerve block experts and has been employed in the investigation of postoperative analgesia after spinal surgery [4, 11, 12]. Because local anesthetics in ESPB can spread to multiple levels [3], ESPB can be conducted not only at the midpoint of the incision but also at the lower thoracic level in lumbar spine surgery. There are three advantages associated with using a low-thoracic ESPB: (1) the target is located outside of the surgical area, which reduces the risk of incision infection; (2) the lumbar ESM is thick, and the middle layer of the thoracolumbar fascia below the ESM is attached to the tip of the lumbar transverse process and the intertransverse process ligaments to form a tenuous connection [13] that is difficult to separate; and (3) when compared to the lumbar ESM, the thoracic muscles are thinner and the transverse process is shallower, making the procedure easier.

Singh et al. [14] reported that by injecting 20 mL of local anesthetics at the T10 transverse process, the skin sensory block range may reach T6-L3, decreasing the need for opioids after lumbar spine surgery and improving patient satisfaction. The twelfth thoracic vertebra is closer to the lumbar spine. In theory, the same volume of local anesthetic diffuses more extensively toward the lumbar spine when injected at this location as when injected at other thoracic levels. Van den Broek et al. [4] conducted a randomized controlled trial in which a 20 mL injection of local anesthetics at the bilateral T12 vertebra significantly reduced the need for postoperative self-controlled analgesia after lumbar spine surgery. We also chose the T12 vertebra for ESPB. The primary outcome revealed that both groups experienced mild pain at 12 h after surgery, with a marginal decrease of 1 point observed in the ESPB group compared to the control group. Although this reduction is statistically significant, its practical significance may be limited. However, the difference in the NRS score between the two groups at 0, 0.5, and 4 h was 3, 3, and 2 points, respectively, surpassing the recommended threshold for a minimal clinically significant change in the pain score [15]. This indicated the efficacy of T12 ESPB as a viable treatment option for acute postoperative pain following lumbar spine surgery. In addition, the median NRS score in the ESPB group was maintained at 0–2 within 72 h after the operation, and the tramadol dosage in the ESPB group was significantly lower than that in the control group.

The concentration and volume of local anesthetics affect the blocking effect of ESPB, but there is no consensus on the optimal concentration and volume of local anesthetics. In two randomized controlled trials, 25 mL of 0.3% ropivacaine [12] and 30 mL of 0.375% ropivacaine [5] were injected into the bilateral transverse processes of the T12 vertebra. Both reduced NRS scores and opioid use after lumbar spine surgery. Our findings showed that a bilateral injection of 20 mL of 0.4% ropivacaine into the T12 transverse process to achieve an ESPB provided adequate postoperative analgesia. The analgesic effect of a 20 mL injection at a lower concentration can be explored in the future.

An ultrasound-guided thoracolumbar interfascial plane (TLIP) block is aimed at the fascia between the lumbar multifidus and longissimus muscles, and local anesthetics are injected to block the medial branch of the posterior branch of the spinal nerve [16]. Bilateral TLIP block can effectively alleviate the pain of lumbar spine surgery [17]. Compared with the TLIP block, T12 ESPB provided better analgesia, lower NRS scores at rest, a lower frequency of PCA compression, and less use of opioids for analgesia [18]. There are two reasons that may weaken the analgesic efficacy of TLIP block: (1) local anesthetics are injected into the thoracolumbar fascia in the operational area during TLIP block, which may be washed during the surgery; (2) the complicated structure of the thoracolumbar fascia impacts the diffusion of local anesthetics. In addition, the target of ESPB is the transverse process, which is easier to identify than the fascia in the TLIP block. Therefore, T12 ESPB may be a more effective peripheral nerve block than TLIP block in lumbar spine surgery.

As a brain complication, POD seriously affects postoperative recovery in older patients, and its occurrence has obvious timing characteristics, generally occurring from 24 to 72 h after surgery [19]. POD has a negative influence on both early and long-term results, with studies revealing a 2–3 fold greater risk of postoperative complications, longer hospital stays and increased medical costs during hospitalization. A retrospective study showed that the incidence of POD in lumbar spine surgery was 0.84% [20]. Elderly patients are more likely to have POD, and the prevalence of POD is notably higher in people older than 65 years of age [21]. In another retrospective study of lumbar spine surgery, researchers found a 14.5% incidence of POD in people older than 65 years of age [22]. The patients in this study were older than 60 years of age, and the incidence of delirium in ESPB group versus control group was 6.7% vs. 9.3% respectively to lie between the reported ratio 0.84% [20] and 14.5% [22] in previous studies. The pathophysiological mechanism of POD is still being explored, and there are many predisposing factors and trigger factors, such as deep anesthesia [23], intraoperative blood pressure fluctuation [24], pain [2] and massive use of opioids [25]. General anesthesia combined with ESPB had lower postoperative pain scores and less opioid use. However, the incidence of POD was not markedly decreased in the ESPB group.

The impact of the regional block on POD is controversial. The meta-analysis performed by Abou-setta et al. [26] provided moderate evidence that a regional block can decrease the risk of delirium in hip fracture patients. However, a 2017 Cochrane meta-analysis of hip fracture patients did not show sufficient high-quality evidence that a regional block reduces the risk of POD [27]. Subsequently, a multicenter randomized controlled trial published in JAMA in 2022 showed that regional anesthesia did not markedly decrease the incidence of POD in patients older than 65 years of age after hip fracture surgery [28]. A study on the pathophysiological mechanism of POD in hip fracture surgery is now being conducted [29]. We believe that these findings will lead to novel approaches to the prevention and treatment of POD.

ESPB is a simple truncal block. Because the transverse process is easy to identify with ultrasound, young physicians with no expertise with ultrasound-guided nerve block can quickly learn the technique for ESPB. Furthermore, serious complications are less likely to occur because the puncture site is far from vital structures such as nerves, blood arteries, and viscera. In this study, there were no complications in the ESPB group, such as puncture site infection, hemorrhage, lumbar plexus block, or nerve injury. Therefore, we recommend promoting the use of T12 ESPB in lumbar spine surgery.

This trial has several limitations. First, ESPB was performed after anesthesia induction and we did not detect the range of the cutaneous sensory block; therefore, patients for who the block failed cannot be excluded. Moreover, the range of nerve block that might be achieved with 20 mL of local anesthetics injected at the T12 vertebral level was not identified. Second, because ESPB is performed without intraoperative blinding of the anesthesiologist, the intraoperative data he collects are likely to be biased. Last, the small sample size of the two groups may be inadequate in detecting differences in the incidence of POD.

## Conclusions

Bilateral ultrasound-guided T12 ESPB appears to be effective regional anesthesia for lumbar spine surgery. It is associated with accelerating recovery in elderly patients after lumbar spine surgery by providing appropriate postoperative analgesia. In the future, it might be used routinely for multimodal analgesia in lumbar spine surgery.

## Data Availability

All data generated or analyzed during this study are included in this published article.

## Abbreviations

CAM: The confusion assessment method
ESPB: Erector spinae plane block
MMSE: Mini-mental state examination
NRS: Numeric rating scales
POD: Postoperative delirium
PONV: Postoperative nausea and vomiting
SAS: Sedation-Agitation Scale
TLIP: Thoracolumbar interfascial plane

## References

[1] Gerbershagen HJ, Aduckathil S, van Wijck AJ (2013). Pain intensity on the first day after Surgery: a prospective cohort study comparing 179. Surg Procedures Anesthesiology.

[2] Gao R, Yang ZZ, Li M (2008). Probable risk factors for postoperative delirium in patients undergoing spinal Surgery. Eur Spine J.

[3] Chin KJ, El-Boghdadly K (2021). Mechanisms of action of the erector spinae plane (ESP) block: a narrative review. Can J Anaesth.

[4] van den Broek RJC, van de Geer R, Schepel NC (2021). Evaluation of adding the Erector Spinae plane block to standard anesthetic care in patients undergoing posterior lumbar interbody fusion Surgery. Sci Rep.

[5] Chen K, Wang L, Liu X (2021). Ultrasound-guided Erector Spinae Plane Block reduces perioperative opioid consumption in lumbar spinal Fusion. Am J Ther.

[6] Zhang TJ, Zhang JJ, Qu ZY (2020). Bilateral Erector Spinae Plane blocks for Open posterior lumbar Surgery. J Pain Res.

[7] Wang Z, Zhang M, Qu G (1989). The application of the Chinese version of Mini Mental State Examination. Shanghai Psychiatry Medicine.

[8] Shim JG, Ryu KH, Kim PO (2020). Evaluation of ultrasound-guided erector spinae plane block for postoperative management of video-assisted thoracoscopic Surgery: a prospective, randomized, controlled clinical trial. J Thorac Dis.

[9] Zou Y, Cole MG, Primeau FJ (1998). Detection and diagnosis of delirium in the elderly: psychiatrist diagnosis, confusion assessment method, or consensus diagnosis?. Int Psychogeriatr.

[10] Forero M, Adhikary SD, Lopez H (2016). The Erector Spinae Plane Block: a novel analgesic technique in thoracic neuropathic Pain. Reg Anesth Pain Med.

[11] Goel VK, Chandramohan M, Murugan C (2021). Clinical efficacy of ultrasound guided bilateral erector spinae block for single-level lumbar fusion Surgery: a prospective, randomized, case-control study. Spine J.

[12] Zhang Q, Wu Y, Ren F (2021). Bilateral ultrasound-guided erector spinae plane block in patients undergoing lumbar spinal fusion: a randomized controlled trial. J Clin Anesth.

[13] Macintosh JE, Bogduk N. 1987 Volvo award in basic science. The morphology of the lumbar erector spinae.Spine (Phila Pa 1976).1987;12(7):658–668. 10.1097/00007632-198709000-00004 3686217

[14] Singh S, Choudhary NK, Lalin D (2020). Bilateral Ultrasound-guided Erector Spinae Plane Block for postoperative analgesia in lumbar spine Surgery: a Randomized Control Trial. J Neurosurg Anesthesiol.

[15] Laigaard J, Pedersen C, Rønsbo TN (2021). Minimal clinically important differences in randomised clinical trials on pain management after total hip and knee arthroplasty: a systematic review. Br J Anaesth.

[16] Hand WR, Taylor JM, Harvey NR (2015). Thoracolumbar interfascial plane (TLIP) block: a pilot study in volunteers. Can J Anaesth.

[17] Ueshima H, Hara E, Otake H (2019). RETRACTED: Thoracolumbar interfascial plane block provides effective perioperative pain relief for patients undergoing lumbar spinal Surgery; a prospective, randomized and double blinded trial. J Clin Anesth.

[18] Wang L, Wu Y, Dou L (2021). Comparison of two Ultrasound-guided Plane blocks for Pain and postoperative opioid requirement in lumbar Spine Fusion Surgery: a prospective, randomized, and controlled clinical trial. Pain Ther.

[19] Evered L, Silbert B, Knopman DS (2018). Recommendations for the nomenclature of cognitive change associated with anaesthesia and surgery-2018. Br J Anaesth.

[20] Fineberg SJ, Nandyala SV, Marquez-Lara A (2013). Incidence and risk factors for postoperative delirium after lumbar spine surgery.Spine. (Phila Pa 1976).

[21] Pandharipande P, Shintani A, Peterson J (2006). Lorazepam is an Independent risk factor for transitioning to delirium in intensive care unit patients. Anesthesiology.

[22] Pan Z, Huang K, Huang W (2019). The risk factors associated with delirium after lumbar spine Surgery in elderly patients. Quant Imaging Med Surg.

[23] Piao J, Jin Y, Lee SM (2018). Triggers and nursing influences on delirium in intensive care units. Nurs Crit Care.

[24] Hirsch J, DePalma G, Tsai TT (2015). Impact of intraoperative hypotension and blood pressure fluctuations on early postoperative delirium after non-cardiac Surgery. Br J Anaesth.

[25] Gaudreau JD, Gagnon P, Roy MA (2007). Opioid medications and longitudinal risk of delirium in hospitalized cancer patients. Cancer.

[26] Abou-Setta AM, Beaupre LA, Rashiq S (2011). Comparative effectiveness of pain management interventions for hip fracture: a systematic review. Ann Intern Med.

[27] Guay J, Parker MJ, Griffiths R (2017). Peripheral nerve blocks for hip fractures.Cochrane database. Syst Rev.

[28] Li T, Li J, Yuan L et al. Effect of Regional vs General Anesthesia on incidence of postoperative delirium in older patients undergoing hip fracture Surgery: the RAGA Randomized Trial.JAMA.2022;327(1):50–8. 10.1001/jama.2021.22647. 10.1001/jama.2021.22647 PMC8689436 34928310

[29] Gamberale R, D’Orlando C, Brunelli S, et al. Study protocol: understanding the pathophysiologic mechanisms underlying delirium in older people undergoing hip fracture Surgery. BMC Geriatr. 2021;21(1). 10.1186/s12877-021-02584-1. 10.1186/s12877-021-02584-1 PMC8567587 34736422

